# A Citation-Based Analysis and Review of Significant Papers on Timing and Time Perception

**DOI:** 10.3389/fnins.2016.00330

**Published:** 2016-07-15

**Authors:** Sundeep Teki

**Affiliations:** Department of Physiology, Anatomy and Genetics, University of OxfordOxford, UK

**Keywords:** timing, time perception, rhythm perception, music perception, interval timing, temporal processing, citations, bibliometrics

## Abstract

Time is an important dimension of brain function, but little is yet known about the underlying cognitive principles and neurobiological mechanisms. The field of timing and time perception has witnessed tremendous growth and multidisciplinary interest in the recent years with the advent of modern neuroimaging and neurophysiological approaches. In this article, I used a data mining approach to analyze the timing literature published by a select group of researchers (*n* = 202) during the period 2000–2015 and highlight important reviews as well as empirical articles that meet the criterion of a minimum of 100 citations. The qualifying articles (*n* = 150) are listed in a table along with key details such as number of citations, names of authors, year and journal of publication as well as a short summary of the findings of each study. The results of such a data-driven approach to literature review not only serve as a useful resource to any researcher interested in timing, but also provides a means to evaluate key papers that have significantly influenced the field and summarize recent progress and popular research trends in the field. Additionally, such analyses provides food for thought about future scientific directions and raises important questions about improving organizational structures to boost open science and progress in the field. I discuss exciting avenues for future research that have the potential to significantly advance our understanding of the neurobiology of timing, and propose the establishment of a new society, the Timing Research Forum, to promote open science and collaborative work within the highly diverse and multidisciplinary community of researchers in the field of timing and time perception.

## Introduction

Natural sounds have a rich temporal structure, in the form of sequences of sounds that rapidly change over time and result in dynamic states of perceptual organization. Natural sound sequences like speech and music form sequences of temporal intervals, often evoking the percept of a rhythm. How the brain processes time intervals and rhythmic sound sequences is an unresolved and challenging problem, given the absence of dedicated neural systems for encoding time.

William James was one of the first psychologists to recognize time as a “sensation,” and heralded a longstanding interest and debate on the nature of time perception and its underlying representation in the brain (James, 1890). William Gooddy, recognized the importance of motor structures for timing from a neurological perspective and suggested that they act as “observers” of time (Gooddy, 1958). Braitenberg (1967) proposed the cerebellum as an internal timekeeper and hypothesized that parallel fibers act as delay lines and provide a means to represent temporal patterns. In the 1970 and 1980s, electrophysiological studies led by Llinas, Cohen and colleagues revealed the specialization of the olivocerebellar circuits for temporal representation (Llinas et al., 1974; Llinás and Yarom, 1981; Welsh et al., 1995; see Yarom and Cohen, 2002 for a review). At the same time, fundamental properties of timing behavior like scalar property provided a theoretical foundation that formal models of an internal clock must address (Church, 1984; Gibbon et al., 1984). In the 1980s and 1990s, neuropsychological work in patients with disorders of the cerebellum and basal ganglia (e.g., Ataxia, Parkinson's) began to provide causal evidence for a role of these brain regions in perceptual and motor timing (Ivry et al., 1988; Ivry and Keele, 1989; Artieda et al., 1992; Pastor et al., 1992; Ivry, 1993; Nichelli et al., 1996).

In the last two decades, however, scientific interest and progress in understanding the neural codes and mechanisms underlying temporal processing has advanced rapidly, aided by technological developments in functional neuroimaging techniques like magnetic resonance imaging and magnetoencephalography; brain stimulation techniques like transcranial magnetic stimulation and transcranial current stimulation; as well as progress in neural recording methods with the development of dense multi-electrode arrays, two-photon calcium imaging, genetic and molecular biology tools including the use of novel experimental animals models and optogenetic targeting of specific cell-types for causal investigations amongst others. Our understanding of the neural mechanisms and circuits involved in temporal computations has significantly advanced through the use of these new technologies and continues to shed light on their underlying brain bases.

However, paralleling the recent advancements in the field is an exponential growth in research output in terms of more research articles, conference proceedings, and new journals. Therefore, unlike in the previous decades, a synthesis of the research advances in the field poses a significant challenge. Discovery of knowledge represents an acute problem with a low “signal-to-noise” threshold, and it is a veritable challenge for a new or even a current investigator in the field to assimilate new ideas and apply these concepts for designing innovative experimental paradigms.

In order to make sense of the progress in the field of timing and time perception in the last fifteen years, I have adopted a data-mining approach to identify key review articles and empirical papers, from a select group of authors that have significantly impacted research on the cognitive and neural principles of time perception. The process involved shortlisting a group of established researchers in the field of timing, and identifying articles published during the period 2000–2015 that have received a minimum of 100 citations. Each qualifying article (*n* = 150) from this group of authors (*n* = 202) is listed in Table 1 along with the number of citations, the rank of each article in terms of number of citations as well as number of citations normalized by time since publication, the names of the authors, the name of the journal, the year of publication, whether the article was an empirical study or a review, and a short summary of each article.

**Table 1:** **List of 150 papers on timing and time perception from 2000 to present sorted according to the number of citations (minimum of 100 citations) in Google Scholar collated on 10 April, 2016 (see Section Key Papers on Timing and Time Perception for More Details)**.

**Citation and rank**	**Reference**	**Year**	**Journal**	**Summary**
1305* [1, 1]	Patel	2008	Oxford Uni Press	A book that analyses music cognition in relation to language from the standpoint of cognitive neuroscience.
1192*, [2, 2]	Buhusi and Meck	2005	*Nat. Rev. Neurosci.*	Time is represented in a distributed manner through coincidental activation of cortico-striatal neuronal populations.
1054, [3, 4]	Boroditsky	2001	*Cogn. Psychol.*	Native language shapes how we think about time.
1036, [4, 6]	Boroditsky	2000	*Cognition*	Time structure is shaped by metaphorical mapping from experiential domains like space.
719, [5, 13]	Rao et al.	2001	*Nat. Neurosci.*	Cortical-subcortical network mediates different components of temporal processing.
623, [6, 3]	Casasanto and Boroditsky	2008	*Cognition*	Spatial information affects judgments about duration but not vice versa.
622*, [7, 14]	Lewis and Miall	2003	*Curr. Opin. Neurobiol.*	Timing is measured by automatic (motor) system and cognitive (prefrontal and parietal) systems.
587*, [8, 12]	Mauk and Buonomano	2004	*Ann. Rev. Neurosci.*	Temporal processing depends on state-dependent changes in network dynamics.
569*, [9, 15]	Matell and Meck	2004	*Cogn. Brain. Res.*	Striatal beat frequency model proposes basal ganglia as coincidence detector of cortical and thalamic input.
551*, [10, 16]	Ivry and Spencer	2004	*Curr. Opin. Neurobiol.*	Cerebellum mediates precise timing and basal ganglia mediates decisions for longer intervals.
512, [11, 11]	Wittmann et al.	2006	*Chronobiol. Int.*	Social jetlag, i.e., the discrepancy between social and biological timing affects wellbeing and stimulant consumption.
469, [12, 10]	Grahn et al.	2007	*J. Cogn. Neurosci.*	Basal ganglia and Supplementary Motor Areas mediate beat perception, in addition to motor production.
450, [13, 23]	Coull et al.	2004	*Science*	Attention to time is mediated by a corticostriatal network.
410*, [14, 45]	Matell and Meck	2000	*Bioessays*	Coincidence detection of neural activity represents a fundamental mechanism of timing.
379*, [15, 47]	Grondin	2001	*Psychol. Bull.*	Weber's law provides a framework for psychological models of time.
364*, [16, 25]	Ivry et al.	2006	*Ann. N. Y. Acad. Sci.*	Cerebellum provides an explicit representation of time.
364, [17, 50]	Coull et al.	2000	*Neuropsychologia*	Temporal orienting depends on sensory events and top-down expectations.
360*, [18, 8]	Grondin	2010	*Att. Percept. Psychophys.*	Review of recent behavioral and neuroscientific studies of timing.
346, [19, 41]	Spencer et al.	2003	*Science*	Cerebellar patients can produce continuous rhythmic movements but not discontinuous movements.
338*, [20, 19]	Ivry and Schlerf	2008	*Trends Cogn. Sci.*	Dedicated models of timing are preferred over intrinsic models.
333, [21, 24]	Karmarkar and Buonomano	2007	*Neuron*	Cortical networks can read out time as a result of intrinsic network dynamics.
332*, [22, 5]	Coull et al.	2011	*Neuropsychopharmacology*	Review of neuroimaging, neuropsychological and psychopharmacological aspects of timing.
320, [23, 21]	Chen et al.	2008	*Cereb. Cortex*	Passively listening to rhythms recruits motor regions of the brain.
318*, [24, 28]	Droit-Volet and Meck	2007	*Trends Cogn. Sci.*	Review of how emotional arousal and valence modulates attentional time-sharing and clock speed.
318, [25, 29]	Shuler and Bear	2006	*Science*	Primary sensory cortex, like V1, mediates reward-timing activity.
315*, [26, 62]	Lewkowicz	2000	*Psychol. Bull.*	Temporal relations emerge in a hierarchical and sequential fashion.
306, [27, 17]	Patel et al.	2009	*Curr. Biol.*	Snowball, a cuckatoo, can spontaneously synchronize its movements to a musical beat.
296, [28, 39]	Morrone et al.	2005	*Nat. Neurosci.*	Short intervals of time between two successive perisaccadic visual stimuli (but not auditory) are underestimated.
289, [29, 51]	Lewis and Miall	2003	*Neuropsychologia*	Distinct brain areas encode time in the sub- and supra-second range.
287*, [30, 26]	Wittmann and Paulus	2008	*Trends Cogn. Sci.*	Review of how impulsivity affects perception of time and decision making.
283, [31, 77]	Penney et al.	2000	*J. Exp. Psychol. Hum. Perc. Perf.*	Attention modulates the internal clock at different rates for auditory and visual signals.
268, [32, 22]	Winkler et al.	2009	*Proc. Natl. Acad. Sci. U.S.A.*	Newborn infants show beat perception.
267, [33, 9]	MacDonald et al.	2011	*Neuron*	Hippocampal time cells encode successive moments during a sequence of events.
257*, [34, 18]	Wiener et al.	2010	*Neuroimage*	Meta analysis that suggests distinct for perceptual vs. motor timing; SMA and right IFG are most commonly activated in various timing tasks.
256*, [35, 71]	Meck and Benson	2002	*Brain Cogn.*	Frontostriatal circuits are involved in interval timing and shifting attention between contexts.
241*, [36, 32]	Meck et al.	2008	*Curr. Opin. Neurobiol.*	Review that proposes striatum serves as a core timer, as part of a distributed timing system.
241*, [37, 40]	Nobre et al.	2007	*Curr. Opin. Neurobiol.*	Review that describes how temporal expectations modulate perception and action, and the underlying neural mechanisms.
241*, [38, 52]	Meck	2005	*Brain Cogn.*	Review of timing that suggests a distributed representation of time across multiple neural systems.
240, [39, 70]	Patel et al.	2003	*Cognition*	Rhythms of French and English speech and music are different.
237*, [40, 35]	Coull and Nobre	2008	*Curr. Opin. Neurobiol.*	Review that suggests basal ganglia is key for explicit timing while parietal and premotor areas mediate implicit timing.
235*, [41, 92]	Nobre	2001	*Neuropsychologia*	Optimization of behavior by temporal orienting is reflected in latency and amplitude of ERPs.
234, [42, 99]	Buonomano	2000	*J. Neurosci.*	Neural circuits possess intrinsic synaptic mechanisms for timing.
231, [43, 83]	Gentner et al.	2002	*Lang. Cogn. Proc.*	Humans use spatial metaphors in temporal reasoning and language.
230, [44, 64]	Vroomen et al.	2004	*Cogn. Brain Res.*	Perception of temporal order is shaped by exposure to audio-visual asynchronies.
222, [45, 89]	Janata et al.	2002	*Cogn. Aff. Behav. Neurosci.*	Attentive listening to music is mediated by domain-general areas.
220, [46, 38]	Chen et al.	2008	*J. Cogn. Neurosci.*	Musicians show greater prefrontal cortex activity vs. non-musicians while tapping to complex auditory rhythms.
218, [47, 82]	Matell et al.	2003	*Behav. Neurosci.*	Striatal and cortical neurons encode time intervals in their firing rates.
213, [48, 109]	Medina et al.	2000	*J. Neurosci.*	Computer simulations show that cerebellum can learn adaptively timed responses.
212*, [49, 42]	Eagleman	2008	*Curr. Opin. Neurobiol.*	Review summarizing illusions of time perception in humans.
208, [50, 65]	Patel et al.	2005	*Exp. Brain. Res.*	Beat perception and synchronization show modality specific benefits for auditory vs. visual beat patterns.
207, [51, 36]	Grahn and Rowe	2009	*J. Neurosci.*	Putamen, SMA and premotor cortex are important for internal generation of the beat and auditory motor coupling during beat perception.
197, [52, 61]	Meck	2006	*Brain Res.*	Dopamine depleting lesions in different parts of the basal ganglia shows dissociable effects on duration discrimination.
197, [53, 122]	Cemgil et al.	2000	*J. New Mus. Res.*	Kalman filter based approach can be used to track tempo.
187*, [54, 67]	Lewis and Miall	2006	*Trends Cogn. Sci.*	Dorsolateral prefrontal cortex mediates working memory as well as timing.
186*, [55, 110]	Buonomano and Karmarkar	2002	*Neuroscientist*	Review that argues that time is coded by the population activity of a large group of neurons.
185, [56, 43]	Arvaniti	2009	*Phonetica*	Review of work on rhythmic categorization which argues that timing is distinct from rhythm.
185, [57, 112]	Buhusi and Meck	2002	*Behav. Neurosci.*	Dopamine modulates attentional components of interval timing.
184*, [58, 7]	Merchant et al.	2013	*Ann. Rev. Neurosci.*	Review that highlights the role of a core timing mechanism in the basal ganglia and its interaction with context dependent areas.
183, [59, 56]	Noesselt et al.	2007	*J. Neurosci.*	Temporal correspondence between auditory and visual streams modulates activity of multisensory STS as well as unisensory cortices.
182*, [60, 31]	Kotz and Schwartze	2010	*Trends Cogn. Sci.*	Review which suggests that temporal and speech processing is processed by cortical and subcortical systems associated with motor control.
182*, [61, 72]	Patel	2006	*Music Percept.*	Review that focuses on the evolutionary aspects of musical rhythm.
179*, [62, 34]	Vroomen and Kreetels	2010	*Att. Percept. Psychophys.*	Review that focuses on intersensory timing and mechanisms that encode intersensory lags.
179, [63, 58]	Burr et al.	2007	*Nat. Neurosci.*	Short visual events are encoded by visual neural mechanisms with localized receptive fields rather than by a centralized supramodal clock.
178, [64, 59]	Wittmann et al.	2007	*Exp. Brain Res.*	Posterior insula mediates delayed gratification of reward while striatum encodes time delay.
178, [65, 74]	McAuley et al.	2006	*J. Exp. Psychol. General*	Event timing profiles for a battery of perceptual-motor timing tasks vary across the life span (4–95 years old).
177, [66, 27]	Boroditsky et al.	2011	*Cognition*	English and Mandarin speakers think about time differently.
177*, [67, 46]	Wittmann	2009	*Phil. Trans. R. Soc. B*	Review that discusses different models of time perception with a particular focus on the insula as a core timer.
177, [68, 76]	Zelaznik et al.	2006	*J. Exp. Psychol. Hum. Perc. Perf.*	Repetitive tapping and drawing movements highlight explicit vs. implicit timing.
175, [69, 101]	Harrington et al.	2004	*Brain*	Motor vs. clock variability in time reproduction and perception tasks does not support a role for cerebellum in timekeeping.
175, [70, 128]	Yarrow et al.	2001	*Nature*	Perceptual fill-in during saccadic suppression underlies the illusion of chronostasis.
174*, [71, 37]	Block et al.	2010	*Acta Psychol.*	Meta analysis that focuses on the effects of cognitive load on prospective and retrospective duration judgments.
174, [72, 80]	Chen et al.	2006	*Neuroimage*	Metrical structure of musical rhythms modulates functional connectivity between auditory and dorsal premotor cortex.
174, [73, 102]	Droit-Volet et al.	2004	*Cogn. Emot.*	The duration of emotional faces is overestimated compared to neutral ones.
174, [74, 108]	Nenadic et al.	2003	*Exp. Brain Res.*	fMRI during a time estimation task shows activation in right putamen.
172*, [75, 123]	Ivry and Richardson	2002	*Brain Cogn.*	A multiple timer model accounts for timing and coordination of repetitive movements.
169*, [76, 20]	Allman and Meck	2012	*Brain*	Review that focuses on distortions of time perception and timed performance in various neurological and psychiatric conditions.
167*, [77, 68]	Taatgen et al.	2007	*Psychol. Rev.*	A time perception model based on adaptive control of thought-rational can explain effects of attention and learning during time estimation.
165, [78, 133]	Burle and Casini	2001	*J. Exp. Psychol. Hum. Perc. Perf.*	Activation and attention have independent effects on timing performance.
163, [79, 118]	McAuley and Jones	2003	*J. Exp. Psychol. Hum. Perc. Perf.*	Timing performance is enhanced when intervals fall on vs. off the beat.
162, [80, 107]	Lewis et al.	2004	*Neuropsychologia*	Brain activity during over-learned tapping varies with temporal complexity of the sequence.
159, [81, 111]	Harrington et al.	2004	*Cogn. Brain Res.*	Event-related fMRI reveals brain areas subserving different aspects of timing.
158, [82, 60]	O'Reilly et al.	2008	*J. Neurosci.*	Posterior cerebellum provides a temporal signal to cortical networks for spatial orienting.
158, [83, 103]	Matlock et al.	2005	*Cogn. Sci.*	Fictive motion influences temporal reasoning.
156, [84, 104]	Doherty et al.	2005	*J. Neurosci.*	Combined spatial and temporal attention lead to enhanced P1 response.
156, [85, 132]	Droit-Volet and Wearden	2002	*Q. J. Exp. Psychol.*	Visual flicker increases the internal clock speed in young children.
155, [86, 125]	Rubia et al.	2003	*J. Abn. Child Psychol.*	Motor timing is impaired in children with ADHD and hyperactivity.
154*, [87, 94]	Correa et al.	2006	*Brain Res.*	Review that focuses on how temporal attention modulates the amplitude and latency of ERPs like N2 and P300 components.
154*, [88, 114]	Rubia and Smith	2004	*Acta Neurobiol.*	Motor timing and time estimation is mediated by common brain networks.
153, [89, 30]	Nozaradan et al.	2011	*J. Neurosci.*	EEG frequency tagging reveals neural entrainment to beat and meter.
152*, [90, 53]	Rubia et al.	2009	*Phil. Trans. R. Soc. B*	Review that suggests that impulsivity in ADHD is related to compromised timing functions and dopamine dysregulation.
151*, [91, 54]	Droit-Volet and Gil	2009	*Phil. Trans. R. Soc. B*	Review that addresses the role of emotional context on timing.
151, [92, 81]	Pariyadath and Eagleman	2007	*PLoS ONE*	Repetition suppression underlies duration distortion.
151*, [94, 117]	Coull	2004	*Cogn. Brain Res.*	Frontal operculum is key for mediating attentional aspects of time estimation.
151, [93, 129]	Desain and Honing	2003	*Perception*	Musical metro primes the perception of rhythmic categories.
150, [95, 33]	Teki et al.	2011	*J. Neurosci.*	Perception of relative and absolute time is mediated by distinct networks based in the basal ganglia and the cerebellum, respectively.
148, [96, 84]	Noulhiane et al.	2007	*Emotion*	Emotional stimuli are judged longer than neutral stimuli, when balanced for the levels of arousal.
147, [97, 98]	Kanai et al.	2006	*J. Vis.*	Temporal frequency of a stimulus serves as the clock for perceived duration.
145, [98, 55]	Kotz et al.	2009	*Cortex*	Review that focuses on the non-motor functions of basal ganglia with particular emphasis on prediction in speech and language.
143, [99, 87]	Styns et al.	2007	*Hum. Mov. Sci.*	Walking speed is modulated by the tempo of musical and metronome stimuli.
140, [100, 49]	Fuhrman and Boroditsky	2010	*Cogn. Sci.*	Temporal judgments in nonlinguistic tasks are influenced by culturally specific spatial representations.
140*, [101, 115]	Eagleman et al.	2005	*J. Neurosci.*	Review of timing based on psychophysics, electrophysiology, imaging and computational modeling.
139, [102, 105]	Lewis and Miall	2006	*Behav. Proc.*	Dorsolateral prefrontal cortex mediates working memory and posterior parietal cortex and anterior cingulate attentional aspects of timing.
137, [103, 106]	Rammsayer and Altenmuller	2006	*Music Percept.*	Musicians perform better than non-musicians in temporal discrimination but not temporal generalization tasks.
136, [104, 63]	Grahn and Brett	2009	*Cortex*	Parkinson's patients show selective deficits in discrimination of beat-based rhythms.
134, [105, 97]	Keller et al.	2007	*Consc. Cogn.*	Action simulation in ensemble musicians like pianists underlies synchronization and self-recognition.
132*, [106, 66]	Eagleman and Pariyadath	2009	*Phil. Trans. R. Soc. B*	Energy expended in coding a stimulus represents its duration.
132, [107, 124]	Navarra et al.	2005	*Cogn. Brain Res.*	Temporal window for audiovisual integration is extended for asynchronous speech and music.
131*, [108, 126]	Lustig et al.	2005	*Memory*	Striatum may detect oscillatory cortical firing in a coincident manner to time brief intervals.
131*, [109, 145]	Mauk et al.	2000	*Curr. Biol.*	Cerebellum is key for movement through feedforward use of sensory information via temporally specific learning.
130, [110, 113]	Patel et al.	2006	*J. Acoust. Soc. Am.*	Music reflects durational patterns in speech as well as patterns of variability in pitch.
130, [111, 135]	Hinton and Meck	2004	*Cogn. Brain Res.*	fMRI activations show involvement of fronto-striatal circuits in interval timing.
127, [112, 88]	Ishihara et al.	2008	*Cortex*	A mental time line exists from left to right along the horizontal axis in space.
127, [113, 116]	Matell et al.	2006	*Psychopharm*	Methamphetamine produces a dose-dependent overestimation of time.
126, [114, 90]	van Eijk et al.	2008	*Att. Percept. Psychophys.*	Synchrony and temporal order judgment tasks produce different PSS estimates.
126, [115, 91]	Wassenhove et al.	2008	*PLoS ONE*	Multisensory interactions influence perception of time: vision can impact auditory temporal perception.
126, [116, 131]	Correa et al.	2005	*Psychon. Bull. Rev.*	Temporal orienting enhances perceptual processing.
125*, [117, 119]	Ivry	2006	*Ann. N. Y. Acad. Sci.*	Review that analyzes the role of the cerebellum as an internal clock.
124, [118, 75]	Iversen et al.	2009	*Ann. N. Y. Acad. Sci.*	Beta-band activity influences auditory rhythm perception.
124, [119, 93]	Wearden et al.	2008	*J. Exp. Psychol. Hum. Perc. Perf.*	Decreasing arousal affects performance on time perception tasks.
123*, [120, 95]	Wearden and Lejeune	2008	*Q. J. Exp. Psychol.*	A review of the conformity and violations of the scalar property in human timing tasks.
123, [121, 138]	Smith et al.	2003	*Neuroimage*	Right dorsolateral prefrontal cortex is involved in time perception, and may serve as an accumulator.
122, [122, 78]	Grahn and McAuley	2009	*Neuroimage*	Individual differences in beat perception exist and modulate activity in auditory and motor areas.
122, [123, 79]	Zarco et al.	2009	*J. Neurophys.*	Performance of rhesus monkeys and humans is compared on a number of sub-second interval reproduction tasks.
121, [124, 136]	Matell et al.	2004	*Behav. Neurosci.*	Intermittent but not continuous administration of cocaine increases the speed of internal clock.
121*, [125, 139]	Wearden	2003	*Time and Mind II*	Book chapter that reviews timing in the light of scalar expectancy theory.
120, [126, 57]	Boroditsky and Gaby	2010	*Psychol. Sci.*	Pormpuraaw, an Australian Aboriginal community represent time according to cardinal directions.
119, [127, 48]	Simen et al.	2011	*J. Neurosci.*	A temporal integration model yields a firing-rate based representation of time.
118, [128, 137]	Correa et al.	2004	*Percept. Psychophys.*	Temporal orienting effects are larger when temporal expectancy is varied between and not within blocks.
118, [129, 144]	Griffin et al.	2002	*Neuropsychologia*	Spatial and temporal orienting optimize behavior through distinct attentional processes.
118, [130, 146]	Droit-Volet and Wearden	2001	*J. Exp. Child Psychol.*	8 year old children show higher temporal sensitivity than 3 and 5 year old children.
117*, [131, 100]	Keller	2008	*Emerg. Comm.*	Review that addresses cognitive processes underlying joint action in music performance.
117, [132, 127]	Vatakis and Spence	2006	*Brain Res.*	Cross-modal temporal discrimination performance is better for audiovisual stimuli of lower complexity.
115, [133, 130]	Effron et al.	2006	*Emotion*	Embodiment plays a role in the emotional modulation of time.
114, [134, 140]	Muller-Gethmann et al.	2003	*Psychophysiol*	Temporal preparation enhances the processing speed of early evoked potentials.
114, [135, 148]	Lustig and Meck	2001	*Psychol. Sci.*	Age-related changes in attentional resources affects interval timing.
113*, [136, 85]	Buhusi and Meck	2009	*Phil. Trans. R. Soc. B*	Attentional and memory resources for timing are shared between timed and intruder events.
112*, [137, 86]	Balsam and Gallistel	2009	*Trends Neurosci.*	Review which suggests that associative learning depends on temporal contiguity.
112, [138, 120]	Droit-Volet et al.	2007	*Behav. Proc.*	5- and 8-year old children underestimate the duration of visual vs. auditory signals.
112, [139, 121]	Stetson et al.	2007	*PLoS ONE*	Slowing of time during threatening events is a function of episodic recollection, not perception.
111, [140, 69]	Casasanto et al.	2010	*Cogn. Sci.*	Spatial information influences temporal judgments more than time affects spatial judgments in children as well as adults.
110, [141, 143]	Lange et al.	2003	*Psychophysiol*	Stimuli presented at attended vs. unattended moments in time yield an enhanced N1 response.
109, [142, 134]	Jahanshahi et al.	2006	*J. Neurosci.*	Basal ganglia and cerebellum are involved in reproduction of both short and long intervals.
108*, [143, 147]	Wing	2002	*Brain Cogn.*	Review that presents an information processing perspective on human voluntary timing.
107, [144, 73]	Jahanshahi et al.	2010	*Brain*	Dopamine increases connectivity between caudate nucleus and prefrontal cortex during motor timing.
106, [145, 96]	Cummins	2009	*J. Phonetics*	Rhythm affords synchronization among two speakers.
104, [146, 44]	Arvaniti	2012	*J. Phonetics*	Rhythm metrics for classification and cross-linguistic comparisons should be used with caution.
104, [147, 141]	Repp and Keller	2004	*Q. J. Exp. Psychol.*	Period correction depends on intention, attention and awareness of tempo changes whilst phase correction depends on intention.
104, [148, 149]	Volz et al.	2001	*Neuroreport*	Schizophrenic patients show hypo-activation in putamen and prefrontal cortex during time estimation.
103*, [149, 142]	MacDonald and Meck	2004	*Neurosci. Biobehav. Rev.*	A review that assesses the close correspondence between reaction time and interval timing.
101*, [150, 150]	Meck	2001	CRC Press	A book that reviews functional and neural mechanisms of interval timing in humans and animals.

Asterisks next to the number of citations denote review articles as opposed to empirical papers. The number of citations, name(s) of authors, year and journal of publication as well as a brief summary is presented for each qualifying article. The authors' names are hyperlinked to the corresponding article's web page on Google Scholar. The numbers in the square brackets next to the number of citations denote the rank of each article in terms of overall number of citations and the rank according to the number of citations normalized by years since publication, respectively. References of all articles in this table are provided in supplementary information.

## Key papers on timing and time perception

To obtain a representative picture of the field, I examined research articles by a select group of experts on timing and time perception. These authors were selected on the basis of their contribution to the recent special issue on “Interval timing and skill learning: the multi sensory representation of Time and Action” published in the Current Opinion of Behavioral Sciences (Meck and Ivry, 2016; 75 authors) as well as on the basis of membership of the recently concluded European COST Action—Timely (http://www.timely-cost.eu/?q=members_list; 127 authors). These 202 authors represented research group all over the world (see Supplementary material B for the complete list of authors), and covered various aspects of timing research including psychophysics, neuroimaging, modeling, and electrophysiology in both humans and experimental animal models.

A number of metrics are commonly used to evaluate the quality and impact of research articles including impact factor, h-index, i-10 index amongst others. Although none of these bibliometrics represent an unbiased estimate of research impact nor are they accepted as standard across the scientific community, the number of citations represents a useful metric as it indicates the impact of a paper and how well the reported findings are accepted and circulated in the field. It is not an ideal measure, for the number of citations an article receives is often skewed by the impact factor of the journal. In order to draw reasonable conclusions about recent progress in the field, articles that were published from 2000 to 2015 and indexed in Google Scholar were considered eligible. Furthermore, to identify the most impactful papers (ideas), a threshold of a minimum of 100 citations was applied. As such a metric may be biased toward older papers than more recent articles, a measure based on the number of citations normalized by the number of years since publication was also considered. Although it is possible to design a more optimal multi-variate measure of research impact (based on number of citations, impact factor of journal or novel altmetrics including number of downloads, number of views and circulation in social media amongst other variables), that is not the motivation of the paper.

Using the above criteria, 150 papers were identified as listed in Table 1 (references of these papers in Supplementary material A; up-to-date as of April 10, 2016). These papers covered topics related to perception of time, rhythm, music, inter-sensory synchrony amongst others and used techniques including psychophysics, neuroimaging, electrophysiology and modeling. Out of the 150 papers, 52 papers were review articles (34.7% of all articles; marked with an asterisk next to the number of citations) that received an average of 271.7 citations (median: 183), i.e., one out of three prominent articles on timing in the last ten years were review articles that either summarized the current state of research or presented new hypotheses to drive the field forward. The remaining empirical papers, 98 in all (65.3% of all articles), received an average of 208 citations per paper (median: 157). Normalizing the number of citations by the number of years since publication to remove the bias due to the “age” of each article revealed a similar trend—review articles receive more citations (mean: 30.0; median: 21.8) than empirical papers (mean: 20.7; median: 16.5). A brief one-sentence summary of each study is also presented in the last column of Table 1, to provide the reader an informed basis to select relevant papers for more in-depth review.

There are several conclusions to be drawn from Table 1, for instance—review articles tend to dominate the field in terms of number of citations while only an average of six significant empirical papers are published every year (also see Supplementary material C, D, and E). Although many of these reviews are now “classic” in the field, even the most recent article in the table is a review (Merchant et al., 2013a; 184 citations). Among other things, this suggests that either the field is still in an embryonic stage where review articles by established researchers are needed to set the precedent on certain topics, or that the field of timing is too diverse, and represents the intersection of various sub-fields including time perception, rhythm perception, music perception, temporal coding, inter sensory asynchrony, motor timing and coordination, that is reflected in the diversity of topics covered by the review articles.

It is not clear whether a similar analysis of the most recent and highly cited papers in other prominent fields like memory, vision, or decision-making will yield similar trends, e.g., ratio of reviews to empirical studies but one could make a null hypothesis that such a ratio may be smaller than for the highly diverse and multidisciplinary field of timing. Alternatively, compared to research topics like vision and memory that have been intensely studied for several decades, the field of timing is still in a nascent stage and does not boast of a large research community as evidenced by the number of specialist journals on timing, or number of exclusive workshops and meetings dedicated to timing research.

## Future directions—scientific

Apart from organizational considerations, there are several new scientific directions that the field can and should embrace to achieve a more comprehensive understanding of the neurobiology of natural timing behavior. Animal models of timing focused on core timing networks including the basal ganglia, cerebellum, premotor and parietal cortex (Grahn, 2012; Schneider and Ghose, 2012; Teki et al., 2012; Merchant et al., 2013a; Allman et al., 2014; Hayashi et al., 2015) will be key to understanding the encoding of time by neuronal ensembles. Such a line of work has been recently pioneered by Merchant and colleagues in rhesus macaques that combines timing behaviors and the examination of the underlying neuronal code in the basal ganglia (Merchant et al., 2011, 2013b; Bartolo et al., 2014; Bartolo and Merchant, 2015). Recent work by Mello et al. (2015) and Gouvêa et al. (2015) further demonstrated that a population code for time exists in the striatum that scales with the interval being timed and multiplexes information about action as well as time. Optogenetic approaches in specific identified cells in animal models will yield crucial insights into the causal role of such mechanisms and their impact on timing behavior (Grosenick et al., 2015). For instance, a recent study by Chen et al. (2014) reported rapid modulation of striatal activity by the cerebellum via a disynaptic pathway that has implications for the coordinated processing of temporal information in these two core timing areas.

The other dominant view of timing is that time is not based on the computations in dedicated circuits but rather represents the output of intrinsic neuronal dynamics (Karmarkar and Buonomano, 2007). In this respect, the activity of sensory areas including auditory, visual, and somatosensory cortices merits further attention. Combining optogenetics and single-unit recordings in primary visual cortex (V1), Hussain Shuler and colleagues have recently provided novel insights into how basal forebrain cholinergic input to V1 provides a teaching signal to modulate the response dynamics of V1 so that cues predictive of given delays to future reward produce responses that express those learned delays (Chubykin et al., 2013; Liu et al., 2015), that those responses reflect learned reward timing (Shuler and Bear, 2006; Zold and Hussain Shuler, 2015) and inform visually-cued timing (Namboodiri et al., 2015). Similar work in other sensory domains such as audition will enable us to decipher the multi-sensory representation of time and action during adaptive behaviors such as speech and movement. Further neurophysiological work using high channel-count electrophysiology (*n* ~ 400–1000) based on new Silicon probes based on CMOS technology (e.g., Berényi et al., 2014; Lopez et al., 2016) or mesoscopic analysis of timing behavior across different cortical layers and multiple brain areas using multi-plane calcium imaging may further shed new light on the underlying circuit-level cortical computations (Yang et al., 2016).

Apart from adopting the latest technological tools and genetic probes, a fundamental understanding of timing can be obtained by designing more naturalistic tasks that use ecological stimuli that are meaningful to the experimental subject in the real world. Naturalistic sequences with variable temporal structure (Teki et al., 2011; Teki and Griffiths, 2014, 2016) that go beyond the traditional use of single intervals may yield novel insights into the encoding of time as well as associated motor behaviors (Kornysheva and Diedrichsen, 2014). Table 1 and the reviews therein highlight that timing is not mediated by a single brain area but rather involves a distributed network (Meck, 2005) in cortical and subcortical areas including prefrontal, parietal, premotor and sensory cortices, insula, basal ganglia, cerebellum, inferior olive amongst others. To formulate a unified theory of how timing is mediated by these structures, it is also important to understand the core functions of these areas and what particular aspect of timing they mediate, whether it is related to perception, attention, or memory. The use of comparative paradigms in healthy human volunteers as well as clinical populations that show timing deficits such as patients with Parkinson's, Huntington's, Schizophrenia amongst others will provide a more uniform understanding of timing functions and dysfunctions in health and disease (Allman and Meck, 2012). An identical approach (and even the use of similar paradigms) in animal models via use of control animals as well as lesion or knock-out models will complement findings from the human literature and provide a more generic understanding of the neural computations and circuits that underlie timing.

## Future directions—organizational

In order to drive more impactful experimental work, the field of timing needs to attract young researchers which would require more concerted efforts from the entire timing community. A recent positive step in this direction was marked by the launch of a specialist journal for timing, Timing and Time Perception (Meck et al., 2013) as well as its corresponding review journal, Timing and Time Perception Reviews. Another step forward would be the launch of an academic society exclusively for researchers in timing that would promote interdisciplinary exchange of ideas amongst researchers with diverse interests in timing via annual conferences that draw on a range of methods from purely behavioral to neurophysiological and neuroanatomical measures; share pertinent news and information like grant funding calls, new papers, job opportunities for doctoral and postdoctoral candidates, workshops and training opportunities; and promote the career development of young researchers through grants for short cross-disciplinary collaborations or exchange visits and funding for attending conferences and mentoring support.

Although there already exist a few scientific societies and communities relevant to timing like the Society for Music Perception and Cognition (SPMC: http://www.musicperception.org), Rhythm Perception and Production Workshop (RPPW: http://rppw.org), European Society for Cognitive Sciences of Music (ESCOM: http://escom2015.org), Society for Education, Music and Psychology Research (SEMPRE: http://www.sempre.org.uk), Deutsche Gesellschaft fur Musikpsychologie (DGM: http://www.music-psychology.de), Asia-Pacific Society for the Cognitive Sciences of Music, Fondazione Mariani (http://fondazione-mariani.org/) that organizes the NeuroMusic conferences, their scope is limited to music perception and psychology, and do not cover all aspects of timing and time perception. Society for Neuroscience (SfN) represents the primary venue where timing researchers gather for structured symposia on human and animal timing research but the scientific discussions are limited given the busy nature of SfN meetings. A recent example of such a successful academic organization for a diverse topic of research is the Society for the Neurobiology of Language (http://www.neurolang.org/) funded by the National Institutes of Health, which since its inception in 2009, attracts more than 400 researchers for its annual conferences. To address the absence of an association of researchers working on all aspects of timing, Argiro Vatakis and I have established a new timing society to promote open science and collaboration—the “Timing Research Forum” (http://timingforum.org).

Irrespective of the present state of affairs, the field of timing and time perception represents a promising and exciting field of research that is growing every year in terms of number of researchers and scientific output, and one where new students and researchers may find a relatively unexplored topic of research and make a significant impact on the field.

## Author contributions

The author confirms being the sole contributor of this work and approved it for publication.

## Funding

ST is funded by the Wellcome Trust (WT106084/Z/14/Z; Sir Henry Wellcome Postdoctoral Fellowship).

### Conflict of interest statement

The author declares that the research was conducted in the absence of any commercial or financial relationships that could be construed as a potential conflict of interest.

## Data

Metrics data presented in Table 1 are available to download as a .mat file from Figshare: Link : https://figshare.com/s/0fb93a59927786300644;DOI : https://dx.doi.org/10.6084/m9.figshare.3153124.
