# Supplementary material for: A Citation-Based Analysis and Review of Significant Papers on Timing and Time Perception
Source: Front Neurosci. 2016 Jul 15;10:330. doi: 10.3389/fnins.2016.00330 (PMC4945625; doi:10.3389/fnins.2016.00330)
Supplement: Supplementary file 1 [file DataSheet1.PDF]

Supplementary material

A citation-based analysis and review of significant papers on timing and time perception

Sundeep Teki

Department of Physiology, Anatomy & Genetics

University of Oxford

South Parks Road

Oxford OX1 3PT, UK

sundeep.teki@dpag.ox.ac.uk

A. References of articles listed in Table 1

1. Allman MJ, Meck WH (2012) Pathophysiological distortions in time perception and timed performance. Brain 135:656–677.
2. Arvaniti A (2009) Rhythm, Timing and the Timing of Rhythm. Phonetica 66:46–63.
3. Arvaniti A (2012) The usefulness of metrics in the quantification of speech rhythm. Journal of Phonetics 40:351–373.
4. Balsam PD, Gallistel CR (2009) Temporal maps and informativeness in associative learning. Trends in Neurosciences 32:73–78.
5. Block RA, Hancock PA, Zakay D (2010) How cognitive load affects duration judgments: A meta-analytic review. Acta Psychologica 134:330–343.
6. Boroditsky L (2000) Metaphoric structuring: Understanding time through spatial metaphors. Cognition 75:1–28.
7. Boroditsky L (2001) Does language shape thought?: Mandarin and English speakers' conceptions of time. Cognitive psychology 43:1–22.
8. Boroditsky L, Fuhrman O, McCormick K (2011) Do English and Mandarin speakers think about time differently? Cognition 118:123–129.
9. Boroditsky L, Gaby A (2010) Remembrances of times east absolute spatial representations of time in an Australian aboriginal community. Psychological Science 21:1635–1639.
10. Buhusi CV, Meck WH (2002) Differential effects of methamphetamine and haloperidol on the control of an internal clock. Behavioral Neuroscience 116:291–297.
11. Buhusi CV, Meck WH (2005) What makes us tick? Functional and neural mechanisms of interval timing. Nature Reviews Neuroscience 6:755–765.
12. Buhusi CV, Meck WH (2009) Relative time sharing: new findings and an extension of the resource allocation model of temporal processing. Philosophical Transactions of the Royal Society of London B: Biological Sciences 364:1875–1885.
13. Buonomano DV (2000) Decoding temporal information: a model based on short-term synaptic plasticity. The Journal of Neuroscience 20:1129–1141.
14. Buonomano DV, Karmarkar UR (2002) Book Review: How Do We Tell Time? The Neuroscientist 8:42–51.

15. Burle B, Casini L (2001) Dissociation between activation and attention effects in time estimation: Implications for internal clock models. Journal of Experimental Psychology: Human Perception and Performance 27:195–205.
16. Burr D, Tozzi A, Morrone MC (2007) Neural mechanisms for timing visual events are spatially selective in real-world coordinates. Nat Neurosci 10:423–425.
17. Casasanto D, Boroditsky L (2008) Time in the mind: Using space to think about time. Cognition 106:579–593.
18. Casasanto D, Fotakopoulou O, Boroditsky L (2010) Space and Time in the Child's Mind: Evidence for a Cross-Dimensional Asymmetry. Cognitive Science 34:387–405.
19. Cemgil AT, Kappen B, Desain P, Honing H (2000) On tempo tracking: Tempogram representation and Kalman filtering. Journal of New Music Research 29:259–273.
20. Chen JL, Penhune VB, Zatorre RJ (2007) Moving on Time: Brain Network for Auditory-Motor Synchronization is Modulated by Rhythm Complexity and Musical Training. Journal of Cognitive Neuroscience 20:226–239.
21. Chen JL, Penhune VB, Zatorre RJ (2008) Listening to Musical Rhythms Recruits Motor Regions of the Brain. Cereb Cortex 18:2844–2854.
22. Chen JL, Zatorre RJ, Penhune VB (2006) Interactions between auditory and dorsal premotor cortex during synchronization to musical rhythms. NeuroImage 32:1771–1781.
23. Correa Á, Lupiáñez J, Madrid E, Tudela P (2006) Temporal attention enhances early visual processing: A review and new evidence from event-related potentials. Brain Research 1076:116–128.
24. Correa Á, Lupiáñez J, Milliken B, Tudela P (2004) Endogenous temporal orienting of attention in detection and discrimination tasks. Perception & Psychophysics 66:264–278.
25. Correa Á, Lupiáñez J, Tudela P (2012) Attentional preparation based on temporal expectancy modulates processing at the perceptual level. Psychonomic Bulletin & Review 12:328–334.
26. Coull JT (2004) fMRI studies of temporal attention: allocating attention within, or towards, time. Cognitive Brain Research 21:216–226.
27. Coull JT, Cheng R-K, Meck WH (2011) Neuroanatomical and neurochemical substrates of timing. Neuropsychopharmacology 36:3–25.

28. Coull JT, Frith CD, Büchel C, Nobre AC (2000) Orienting attention in time: behavioural and neuroanatomical distinction between exogenous and endogenous shifts. Neuropsychologia 38:808–819.
29. Coull JT, Nobre AC (2008) Dissociating explicit timing from temporal expectation with fMRI. Current opinion in neurobiology 18:137–144.
30. Coull JT, Vidal F, Nazarian B, Macar F (2004) Functional anatomy of the attentional modulation of time estimation. Science 303:1506–1508.
31. Cummins F (2009) Rhythm as entrainment: The case of synchronous speech. Journal of Phonetics 37:16–28.
32. Desain P, Honing H (2003) The formation of rhythmic categories and metric priming. Perception 32:341–365.
33. Doherty JR, Rao A, Mesulam MM, Nobre AC (2005) Synergistic effect of combined temporal and spatial expectations on visual attention. The Journal of neuroscience 25:8259–8266.
34. Droit-Volet S, Brunot S, Niedenthal PM (2004) Perception of the duration of emotional events. Cognition and Emotion 18:849–858.
35. Droit-Volet S, Gil S (2009) The time–emotion paradox. Philosophical Transactions of the Royal Society of London B: Biological Sciences 364:1943–1953.
36. Droit-Volet S, Meck WH (2007) How emotions colour our perception of time. Trends in Cognitive Sciences 11:504–513.
37. Droit-Volet S, Meck WH, Penney TB (2007) Sensory modality and time perception in children and adults. Behavioural Processes 74:244–250.
38. Droit-Volet S, Wearden J (2002) Speeding up an internal clock in children? Effects of visual flicker on subjective duration. The Quarterly Journal of Experimental Psychology Section B 55:193–211.
39. Droit-Volet S, Wearden JH (2001) Temporal Bisection in Children. Journal of Experimental Child Psychology 80:142–159.
40. Eagleman DM (2008) Human time perception and its illusions. Current opinion in neurobiology 18:131–136.

41. Eagleman DM, Pariyadath V (2009) Is subjective duration a signature of coding efficiency? Philosophical Transactions of the Royal Society of London B: Biological Sciences 364:1841–1851.

42. Eagleman DM, Tse PU, Buonomano D, Janssen P, Nobre AC, Holcombe AO (2005) Time and the Brain: How Subjective Time Relates to Neural Time. J Neurosci 25:10369–10371.

43. Effron DA, Niedenthal PM, Gil S, Droit-Volet S (2006) Embodied temporal perception of emotion. Emotion 6:1–9.

44. Fuhrman O, Boroditsky L (2010) Cross-Cultural Differences in Mental Representations of Time: Evidence From an Implicit Nonlinguistic Task. Cognitive Science 34:1430–1451.

45. Gentner D, Imai M, Boroditsky L (2002) As time goes by: Evidence for two systems in processing space→ time metaphors. Language and cognitive processes 17:537–565.

46. Grahn JA, Brett M (2007) Rhythm and beat perception in motor areas of the brain. Cognitive Neuroscience, Journal of 19:893–906.

47. Grahn JA, Brett M (2009) Impairment of beat-based rhythm discrimination in Parkinson's disease. Cortex 45:54–61.

48. Grahn JA, McAuley JD (2009) Neural bases of individual differences in beat perception. NeuroImage 47:1894–1903.

49. Grahn JA, Rowe JB (2009) Feeling the beat: premotor and striatal interactions in musicians and nonmusicians during beat perception. The Journal of Neuroscience 29:7540–7548.

50. Griffin IC, Miniussi C, Nobre AC (2002) Multiple mechanisms of selective attention: differential modulation of stimulus processing by attention to space or time. Neuropsychologia 40:2325–2340.

51. Grondin S (2001) From physical time to the first and second moments of psychological time. Psychological Bulletin 127:22–44.

52. Grondin S (2010) Timing and time perception: A review of recent behavioral and neuroscience findings and theoretical directions. Attention, Perception, & Psychophysics 72:561–582.

53. Harrington DL, Boyd LA, Mayer AR, Sheltraw DM, Lee RR, Huang M, Rao SM (2004a) Neural representation of interval encoding and decision making. Cognitive Brain Research 21:193–205.

54. Harrington DL, Lee RR, Boyd LA, Rapcsak SZ, Knight RT (2004b) Does the representation of time depend on the cerebellum? Brain 127:561–574.
55. Hinton SC, Meck WH (2004) Frontal–striatal circuitry activated by human peak-interval timing in the supra-seconds range. Cognitive Brain Research 21:171–182.
56. Ishihara M, Keller PE, Rossetti Y, Prinz W (2008) Horizontal spatial representations of time: Evidence for the STEARC effect. Cortex 44:454–461.
57. Iversen JR, Repp BH, Patel AD (2009) Top-Down Control of Rhythm Perception Modulates Early Auditory Responses. Annals of the New York Academy of Sciences 1169:58–73.
58. Ivry R (2006) Cerebellar Involvement in the Explicit Representation of Temporal Information a. Annals of the New York Academy of Sciences 682:214–230.
59. Ivry RB, Richardson TC (2002) Temporal control and coordination: The multiple timer model. Brain and Cognition 48:117–132.
60. Ivry RB, Schlerf JE (2008) Dedicated and intrinsic models of time perception. Trends in cognitive sciences 12:273–280.
61. Ivry RB, Spencer RM (2004) The neural representation of time. Current opinion in neurobiology 14:225–232.
62. Ivry RB, Spencer RM, Zelaznik HN, Diedrichsen J (2006) The cerebellum and event timing. Annals of the New York Academy of Sciences 978:302–317.
63. Jahanshahi M, Jones CRG, Dirnberger G, Frith CD (2006) The Substantia Nigra Pars Compacta and Temporal Processing. J Neurosci 26:12266–12273.
64. Jahanshahi M, Jones CRG, Zijlmans J, Katzenschlager R, Lee L, Quinn N, Frith CD, Lees AJ (2010) Dopaminergic modulation of striato-frontal connectivity during motor timing in Parkinson's disease. Brain 133:727–745.
65. Janata P, Tillmann B, Bharucha JJ (2002) Listening to polyphonic music recruits domain-general attention and working memory circuits. Cognitive, Affective, & Behavioral Neuroscience 2:121–140.
66. Kanai R, Paffen CLE, Hogendoorn H, Verstraten FAJ (2006) Time dilation in dynamic visual display. Journal of Vision 6:8–8.
67. Karmarkar UR, Buonomano DV (2007) Timing in the absence of clocks: encoding time in neural network states. Neuron 53:427–438.

68. Keller PE (2008) Joint Action in Music Performance. Emerging Communication: Studies in New Technologies and Practices in Communication 14: 205-221. IOS Press Ebooks Volume 10: Enacting Intersubjectivity
69. Keller PE, Knoblich G, Repp BH (2007) Pianists duet better when they play with themselves: On the possible role of action simulation in synchronization. Consciousness and Cognition 16:102–111.
70. Kotz SA, Schwartze M (2010) Cortical speech processing unplugged: a timely subcortico-cortical framework. Trends in Cognitive Sciences 14:392–399.
71. Kotz SA, Schwartze M, Schmidt-Kassow M (2009) Non-motor basal ganglia functions: A review and proposal for a model of sensory predictability in auditory language perception. Cortex 45:982–990.
72. Lange K, Rösler F, Röder B (2003) Early processing stages are modulated when auditory stimuli are presented at an attended moment in time: An event-related potential study. Psychophysiology 40:806–817.
73. Lewis PA, Miall RC (2003a) Distinct systems for automatic and cognitively controlled time measurement: evidence from neuroimaging. Current Opinion in Neurobiology 13:250–255.
74. Lewis PA, Miall RC (2003b) Brain activation patterns during measurement of sub-and supra-second intervals. Neuropsychologia 41:1583–1592.
75. Lewis PA, Miall RC (2006a) Remembering the time: a continuous clock. Trends in Cognitive Sciences 10:401–406.
76. Lewis PA, Miall RC (2006b) A right hemispheric prefrontal system for cognitive time measurement. Behavioural Processes 71:226–234.
77. Lewis PA, Wing AM, Pope PA, Praamstra P, Miall RC (2004) Brain activity correlates differentially with increasing temporal complexity of rhythms during initialisation, synchronisation, and continuation phases of paced finger tapping. Neuropsychologia 42:1301–1312.
78. Lewkowicz DJ (2000) The development of intersensory temporal perception: an epigenetic systems/limitations view. Psychological bulletin 126:281.
79. Lustig C, Matell MS, Meck WH (2005) Not "just" a coincidence: Frontal-striatal interactions in working memory and interval timing. Memory 13:441–448.

80. Lustig C, Meck WH (2001) Paying attention to time as one gets older. Psychological Science 12:478–484.
81. MacDonald CJ, Lepage KQ, Eden UT, Eichenbaum H (2011) Hippocampal "Time Cells" Bridge the Gap in Memory for Discontiguous Events. Neuron 71:737–749.
82. MacDonald CJ, Meck WH (2004) Systems-level integration of interval timing and reaction time. Neuroscience & Biobehavioral Reviews 28:747–769.
83. Matell MS, Bateson M, Meck WH (2006) Single-trials analyses demonstrate that increases in clock speed contribute to the methamphetamine-induced horizontal shifts in peak-interval timing functions. Psychopharmacology 188:201–212.
84. Matell MS, King GR, Meck WH (2004) Differential modulation of clock speed by the administration of intermittent versus continuous cocaine. Behavioral Neuroscience 118:150–156.
85. Matell MS, Meck WH (2000) Neuropsychological mechanisms of interval timing behavior. BioEssays 22:94–103.
86. Matell MS, Meck WH (2004) Cortico-striatal circuits and interval timing: Coincidence detection of oscillatory processes. Cognitive Brain Research 21:139–170.
87. Matell MS, Meck WH, Nicolelis MA (2003) Interval timing and the encoding of signal duration by ensembles of cortical and striatal neurons. Behavioral Neuroscience 117:760–773.
88. Matlock T, Ramscar M, Boroditsky L (2005) On the experiential link between spatial and temporal language. Cognitive science 29:655–664.
89. Mauk MD, Buonomano DV (2004) The neural basis of temporal processing. Annu Rev Neurosci 27:307–340.
90. Mauk MD, Medina JF, Nores WL, Ohyama T (2000) Cerebellar function: coordination, learning or timing? Current Biology 10:R522–R525.
91. McAuley JD, Jones MR (2003) Modeling effects of rhythmic context on perceived duration: a comparison of interval and entrainment approaches to short-interval timing. Journal of Experimental Psychology: Human Perception and Performance 29:1102.
92. McAuley JD, Jones MR, Holub S, Johnston HM, Miller NS (2006) The time of our lives: Life span development of timing and event tracking. Journal of Experimental Psychology: General 135:348–367.

93. Meck WH (2003) Functional and neural mechanisms of interval timing. CRC Press.
94. Meck WH (2005) Neuropsychology of timing and time perception. Brain and Cognition 58:1–8.
95. Meck WH (2006) Neuroanatomical localization of an internal clock: A functional link between mesolimbic, nigrostriatal, and mesocortical dopaminergic systems. Brain Research 1109:93–107.
96. Meck WH, Benson AM (2002) Dissecting the brain's internal clock: How frontal–striatal circuitry keeps time and shifts attention. Brain and Cognition 48:195–211.
97. Meck WH, Penney TB, Pouthas V (2008) Cortico-striatal representation of time in animals and humans. Current Opinion in Neurobiology 18:145–152.
98. Medina JF, Garcia KS, Nores WL, Taylor NM, Mauk MD (2000) Timing mechanisms in the cerebellum: testing predictions of a large-scale computer simulation. The Journal of Neuroscience 20:5516–5525.
99. Merchant H, Harrington DL, Meck WH (2013) Neural basis of the perception and estimation of time. Annual Review of Neuroscience 36:313–336.
100. Morrone MC, Ross J, Burr D (2005) Saccadic eye movements cause compression of time as well as space. Nat Neurosci 8:950–954.
101. MÜller-Gethmann H, Ulrich R, Rinkenauer G (2003) Locus of the effect of temporal preparation: Evidence from the lateralized readiness potential. Psychophysiology 40:597–611.
102. Navarra J, Vatakis A, Zampini M, Soto-Faraco S, Humphreys W, Spence C (2005) Exposure to asynchronous audiovisual speech extends the temporal window for audiovisual integration. Cognitive Brain Research 25:499–507.
103. Nenadic I, Gaser C, Volz H-P, Rammsayer T, Häger F, Sauer H (2003) Processing of temporal information and the basal ganglia: new evidence from fMRI. Exp Brain Res 148:238–246.
104. Nobre AC (2001) Orienting attention to instants in time. Neuropsychologia 39:1317–1328.
105. Nobre AC, Correa A, Coull JT (2007) The hazards of time. Current opinion in neurobiology 17:465–470.
106. Noesselt T, Rieger JW, Schoenfeld MA, Kanowski M, Hinrichs H, Heinze H-J, Driver J (2007) Audiovisual Temporal Correspondence Modulates Human Multisensory Superior Temporal Sulcus Plus Primary Sensory Cortices. J Neurosci 27:11431–11441.

107. Noulhiane M, Mella N, Samson S, Ragot R, Pouthas V (2007) How emotional auditory stimuli modulate time perception. Emotion 7:697–704.

108. Nozaradan S, Peretz I, Missal M, Mouraux A (2011) Tagging the Neuronal Entrainment to Beat and Meter. J Neurosci 31:10234–10240.

109. O'Reilly JX, Mesulam MM, Nobre AC (2008) The cerebellum predicts the timing of perceptual events. The Journal of Neuroscience 28:2252–2260.

110. Pariyadath V, Eagleman D (2007) The effect of predictability on subjective duration. PloS one 2:e1264.

111. Patel AD (2006) Musical rhythm, linguistic rhythm, and human evolution. Music Perception 24:99–104.

112. Patel AD (2008) Music, language, and the brain. Oxford university press.

113. Patel AD, Daniele JR (2003) An empirical comparison of rhythm in language and music. Cognition 87:B35–B45.

114. Patel AD, Iversen JR, Bregman MR, Schulz I (2009) Experimental evidence for synchronization to a musical beat in a nonhuman animal. Current biology 19:827–830.

115. Patel AD, Iversen JR, Chen Y, Repp BH (2005) The influence of metricality and modality on synchronization with a beat. Exp Brain Res 163:226–238.

116. Patel AD, Iversen JR, Rosenberg JC (2006) Comparing the rhythm and melody of speech and music: The case of British English and French. The Journal of the Acoustical Society of America 119:3034–3047.

117. Penney TB, Gibbon J, Meck WH (2000) Differential effects of auditory and visual signals on clock speed and temporal memory. Journal of Experimental Psychology: Human Perception and Performance 26:1770–1787.

118. Rammsayer T, Altenmüller E (2006) Temporal Information Processing in Musicians and Nonmusicians. Music Perception: An Interdisciplinary Journal 24:37–48.

119. Rao SM, Mayer AR, Harrington DL (2001) The evolution of brain activation during temporal processing. Nat Neurosci 4:317–323.

120. Repp BH, Keller PE (2004) Adaptation to tempo changes in sensorimotor synchronization: Effects of intention, attention, and awareness. Quarterly Journal of Experimental Psychology Section A 57:499–521.

121. Rubia K, Halari R, Christakou A, Taylor E (2009) Impulsiveness as a timing disturbance: neurocognitive abnormalities in attention-deficit hyperactivity disorder during temporal processes and normalization with methylphenidate. Philosophical Transactions of the Royal Society of London B: Biological Sciences 364:1919–1931.

122. Rubia K, Noorloos J, Smith A, Gunning B, Sergeant J (2003) Motor timing deficits in community and clinical boys with hyperactive behavior: the effect of methylphenidate on motor timing. Journal of abnormal child psychology 31:301–313.

123. Rubia K, Smith A (2004) The neural correlates of cognitive time management: a review. Acta neurobiologiae experimentalis 64:329–340.

124. Shuler MG, Bear MF (2006) Reward timing in the primary visual cortex. Science 311:1606–1609.

125. Simen P, Balci F, de Souza L, Cohen JD, Holmes P (2011) A model of interval timing by neural integration. The Journal of Neuroscience 31:9238–9253.

126. Smith A, Taylor E, Lidzba K, Rubia K (2003) A right hemispheric frontocerebellar network for time discrimination of several hundreds of milliseconds. Neuroimage 20:344–350.

127. Spencer R, Zelaznik HN, Diedrichsen J, Ivry RB (2003) Disrupted timing of discontinuous but not continuous movements by cerebellar lesions. Science Signalling 300:1437.

128. Stetson C, Fiesta MP, Eagleman DM (2007) Does time really slow down during a frightening event? PLoS One 2:e1295.

129. Styns F, van Noorden L, Moelants D, Leman M (2007) Walking on music. Human Movement Science 26:769–785.

130. Taatgen NA, van Rijn H, Anderson J (2007) An integrated theory of prospective time interval estimation: The role of cognition, attention, and learning. Psychological Review 114:577–598.

131. Teki S, Grube M, Kumar S, Griffiths TD (2011) Distinct Neural Substrates of Duration-Based and Beat-Based Auditory Timing. J Neurosci 31:3805–3812.

132. van Eijk RLJ, Kohlrausch A, Juola JF, Par S van de (2008) Audiovisual synchrony and temporal order judgments: Effects of experimental method and stimulus type. Perception & Psychophysics 70:955–968.

133. Vatakis A, Spence C (2006) Audiovisual synchrony perception for music, speech, and object actions. Brain Research 1111:134–142.

134.Volz HP, Nenadic I, Gaser C, Rammsayer T, Häger F, Sauer H (2001) Time estimation in schizophrenia: an fMRI study at adjusted levels of difficulty. Neuroreport 12:313–316.

135.Vroomen J, Keetels M (2010) Perception of intersensory synchrony: A tutorial review. Attention, Perception, & Psychophysics 72:871–884.

136.Vroomen J, Keetels M, de Gelder B, Bertelson P (2004) Recalibration of temporal order perception by exposure to audio-visual asynchrony. Cognitive Brain Research 22:32–35.

137.Wassenhove V van, Buonomano DV, Shimojo S, Shams L (2008) Distortions of Subjective Time Perception Within and Across Senses. PLOS ONE 3:e1437.

138.Wearden JH (2003) Applying the scalar timing model to human time psychology: Progress and challenges. Time and mind II: Information processing perspectives:21–39.

139.Wearden JH, Lejeune H (2008) Scalar properties in human timing: Conformity and violations. The Quarterly Journal of Experimental Psychology 61:569–587.

140.Wearden JH, Norton R, Martin S, Montford-Bebb O (2007) Internal clock processes and the filled-duration illusion. Journal of Experimental Psychology: Human Perception and Performance 33:716.

141.Wiener M, Turkeltaub P, Coslett HB (2010) The image of time: A voxel-wise meta-analysis. NeuroImage 49:1728–1740.

142.Wing AM (2002) Voluntary timing and brain function: an information processing approach. Brain and cognition 48:7–30.

143.Winkler I, Háden GP, Ladinig O, Sziller I, Honing H (2009) Newborn infants detect the beat in music. PNAS 106:2468–2471.

144.Wittmann M (2009) The inner experience of time. Philosophical Transactions of the Royal Society of London B: Biological Sciences 364:1955–1967.

145.Wittmann M, Dinich J, Merrow M, Roenneberg T (2006) Social Jetlag: Misalignment of Biological and Social Time. Chronobiology International 23:497–509.

146.Wittmann M, Leland DS, Paulus MP (2007) Time and decision making: differential contribution of the posterior insular cortex and the striatum during a delay discounting task. Exp Brain Res 179:643–653.

147.Wittmann M, Paulus MP (2008) Decision making, impulsivity and time perception. Trends in Cognitive Sciences 12:7–12.

148. Yarrow K, Haggard P, Heal R, Brown P, Rothwell JC (2001) Illusory perceptions of space and time preserve cross-saccadic perceptual continuity. Nature 414:302–305.

149. Zarco W, Merchant H, Prado L, Mendez JC (2009) Subsecond Timing in Primates: Comparison of Interval Production Between Human Subjects and Rhesus Monkeys. Journal of Neurophysiology 102:3191–3202.

150. Zelaznik HN, Spencer R, Ivry RB (2002) Dissociation of explicit and implicit timing in repetitive tapping and drawing movements. Journal of Experimental Psychology: Human Perception and Performance 28:575

B. Group of authors considered for analysis of citations

1. Agnieszka Wykowska
2. Åke Hellström
3. Alan Wing
4. Alex Kacelnik
5. Alice Tomassini
6. Amalia Arvaniti
7. Ana Gheorghiu
8. Andréia Kroger
9. Angel Correa
10. Anna Eisler
11. Anna Lambrechts
12. Anne Giersch
13. Anne Reboul
14. Anne-Marie Mouly
15. Anne-Sophie Nyssen
16. Argiro Vatakis
17. Armando Machado
18. Armin Kohlrausch
19. Aurelio Bruno
20. Ayelet Landau

21. Barbara Lewandowska-Tomaszczyk
22. Barbara Tillmann
23. Barry Dantean
24. Beatrice de Gelder
25. Benjamin DeCorte
26. Boris Burle
27. Brigitte Roeder
28. Bruce L. Brown
29. Bruno Moelder
30. Carlos Montemayor
31. Carmel Levitan
32. Catalin Buhusi
33. Catherine Jones
34. Charlotte C. Burn
35. Chris MacDonald
36. Christian S. Jensen
37. Christine Falter
38. Daniel Casasanto
39. Daniel Durstewitz
40. Daniel Linares
41. David Burr
42. David Eagleman
43. David Freestone
44. David J. Lewkowicz
45. David Whitaker
46. Daya Gupta
47. Dean Buonomano
48. Deborah Harrington
49. Denis Mareschal
50. Denis O'Hora
51. Devin Blair Terhune

52. Dmitry Sherbina
53. Eleni Orfanidou
54. Elias Giannopoulos
55. Elijah Petter
56. Elżbieta Szelag
57. Emma Birkett
58. Esposito Anna
59. Fabio Babiloni
60. Ferran Pons
61. Franck Vidal
62. Fred Cummins
63. Fuat Balci
64. Gaëtan Garraux
65. Georges Dellatolas
66. Georgios Papadelis
67. Germund Hesslow
68. Guido Marco Cicchini
69. Guy van Orden
70. Hannes Eisler
71. Hedderik van Rijn
72. Heidi Kloos
73. Henkjan Honing
74. Hugo Merchant
75. Ian Phillips
76. Ignasi Cos
77. Ingrid Scharlau
78. J. Devin McAuley
79. Javier Medina
80. Jean Vroomen
81. Jennifer Coull
82. Jeremie Jozefowiez

83. Jeremy Grivel
84. Jeroen Smeets
85. Jess Hartcher-O'Brien
86. Jessica Lake
87. Jiri Wackermann
88. Joachim Hass
89. Joan López-Moliner
90. Joel Talcott
91. John A. Michon
92. John Iversen
93. John Wearden
94. Joseph Glicksohn
95. Karin Bausenhart
96. Karin Petrini
97. Katarzyna J. Blinowska
98. Katharina Weiß
99. Kathrin Lange
100. Katya Rubia
101. Kia Nobre
102. Kielan Yarrow
103. Kimberly Kirkpatrick
104. Konstantinos Pastiadis
105. Lars Boenke
106. Laurence Schneider
107. Leon van Noorden
108. Lera Boroditsky
109. Lihan Chen
110. Lucas Spierer
111. Marc Ernst
112. Marc Sommer
113. Maren Schmidt-Kassow

114.Maria Giagkou

115.Marieke Rohde

116.Marjan Jahanshahi

117.Mark Elliott

118.Mark Glennon

119.Markus Conci

120.Marshall Shuler

121.Martin Wiener

122.Martine Turgeon

123.Masami Ishihara

124.Massimiliano Di Luca

125.Matthew Matell

126.Melissa Allman

127.Michael Riley

128.Michail Maniadakis

129.Michelle Phillips

130.Mingbo Cai

131.Mona Buhusi

132.Nandakumar Narayanan

133.Narayanan Srinivasan

134.Nathalie Mella

135.Nicholas Lusk

136.Nicla Rossini

137.Nicola S. Clayton

138.Niels Taatgen

139.Niko Busch

140.Olga Pollatos

141.Panagiotis Simos

142.Panos Trahanias

143.Paolo Nichelli

144.Patricia Agostino

145.Patrick Simen

146.Penelope Lewis

147.Peter Balsam

148.Peter Hancock

149.Peter Keller

150.Peter Øhrstrøm

151.Petra Wagner

152.Philippe Palanque

153.Pierre Meyrand

154.Rafael Munoz

155.Ramanujan Raghavan

156.Rattat, Anne-Claire

157.Richard Ivry

158.Robert French

159.Roberta Cermisoni

160.Roberto Aguirre Fernández de Lara

161.Roberto Bottini

162.Rolf Ulrich

163.Rossana Actis

164.Ruey-Kuang Cheng

165.Russell Church

166.Ryota Kanai

167.Salvador Soto Faraco

168.Sean Power

169.Sebastian Wallot

170.Selma Supek

171.Sharon Gilaie-Dotan

172.Simon Grondin

173.Sonja Kotz

174.Sophie Herbst

175.Sorinel Oprisan

176.Stanislava Antonijevic

177.Sundeep Teki

178.Sylvie Droit-Volet

179.Tadeusz Kononowicz

180.Thanos Fouloulis

181.Thomas Ploug

182.Thomas Rammsayer

183.Tiaza Bem

184.Timothy Griffiths

185.Toemme Noesselt

186.Trevor Penney

187.Valdas Noreika

188.Valérie Doyère

189.Valtteri Arstila

190.Vassilis Angelis

191.Vincent C. Müller

192.Vincent Prevosto

193.Virginie van Wassenhove

194.Warren Meck

195.Warrick Roseboom

196.William Hetrick

197.William Matthews

198.Wolfgang Tschacher

199.Yuko Yotsumoto

200.Yvonne Delevoye-Turrell

201.Yvonne Foerster-Beuthan

202.Zhunaghua Shi

C. List of authors ranked according to number of papers (minimum: 3)

Meck WH	23
Boroditsky L	9
Nobre AC	8
Droit-Volet S	7
Ivry RB	7
Patel AD	7
Coull JT	6
Buonomano DV	6
Matell M	6
Lewis PA	5
Wearden JH	5
Miall RC	5
Eagleman D	5
Repp BH	4
Grahn JA	4
Wittmann M	4
Iversen JR	4
Correa A	4
Spencer RM	4
Rubia K	4
Harrington DL	4
Keller PE	4
Chen JL	3
Penhune VB	3
Rao SM	3
Penney TB	3
Zelaznik HN	3
Rammsayer T	3
Honing H	3

Tudela P	3
McAuley JD	3
Mauk MD	3
Zatorre RJ	3
Buhusi CV	3
Frith CD	3
Lupiáñez J	3
Smith A	3

D. Number of papers in Table 1 vs. year of publication

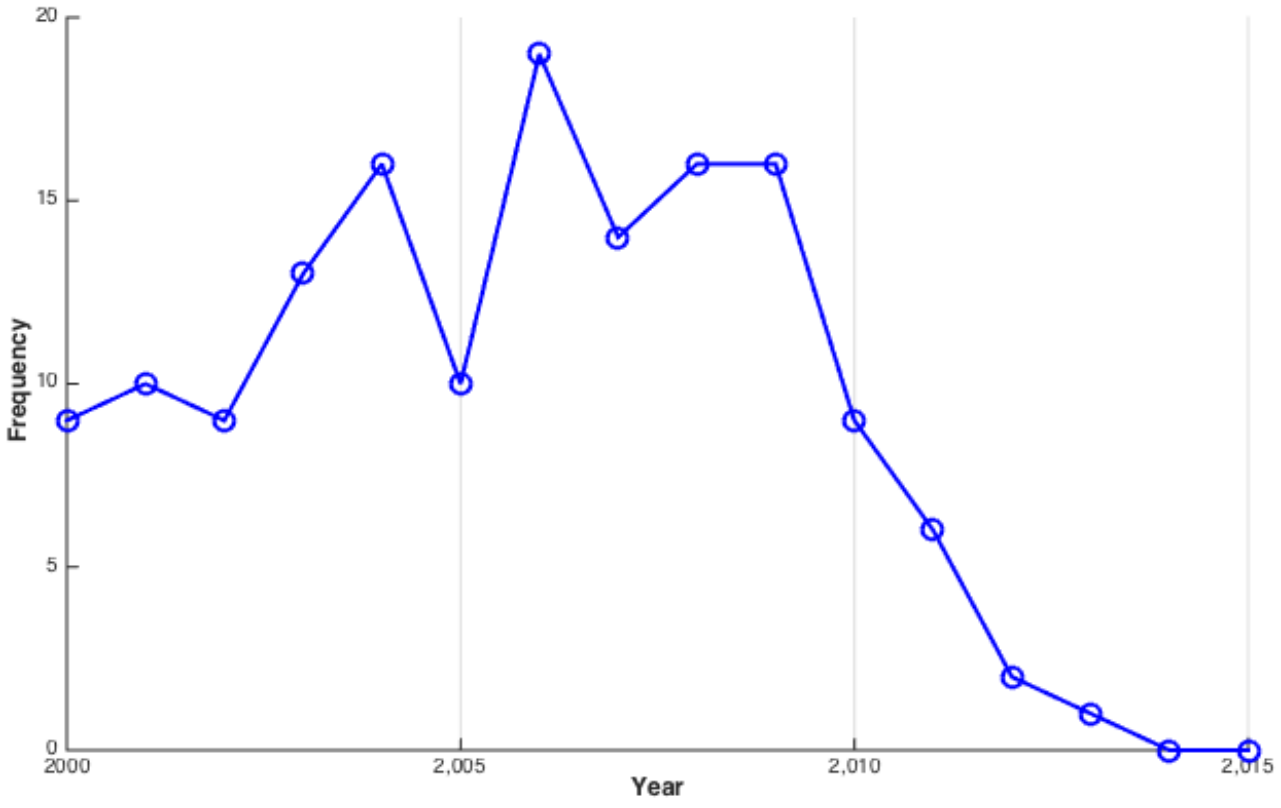

E. List of journals, their frequency and impact factors (2014-15)

Journal	Frequency	Impact factor
Acta Neurobiol	1	1.29
Acta Psychol	1	2.25
Ann NY Acad Sci	3	4.38
Ann Rev Neurosci	2	19.32
Att Percept Psychophys	3	2.17
Behav Neurosci	3	2.73
Behav Proc	2	1.57
BioEssays	1	4.73
Brain	3	9.20
Brain & Cognition	4	2.48
Brain Res	3	2.84
Cereb Cortex	1	8.67
Chronobiol Int	1	3.34
Cogn Aff Behav Neurosci	1	3.29
Cogn Brain Res	6	3.77
Cogn Psychol	1	5.06
Cogn Sci	3	2.38
Cognition	4	3.48
Cognition & Emotion	1	2.52
Consc & Cogn	1	2.31
Cortex	3	5.13
CRC Press	1	-
Curr Biol	2	9.57
Curr Opin Neurobiol	6	6.63
Emerg Comm	1	-

Journal	Frequency	Impact factor
Emotion	2	3.88
Exp Brain Res	3	2.04
Hum Mov Sci	1	1.60
J Abn Child Psychol	1	3.48
J Acoust Soc Am	1	1.50
J Cogn Neurosci	1	4.09
J Exp Child Psychol	1	3.12
J Exp Psychol: General	1	5.50
J Exp Psychol: Hum Perc Perf	5	3.36
J Neurophys	1	2.89
J Neurosci	11	6.34
J New Mus Res	1	0.57
J Phonetics	2	1.41
J Vis	1	2.39
Lang & Cogn Proc	1	1.54
Memory	1	2.09
Music Perception	2	1.63
Nat Neurosci	3	16.10
Nat Rev Neurosci	1	31.43
Nature	1	41.46
NeuroImage	4	6.36
Neuron	2	15.05
Neuropsychologia	5	3.30
Neuropsychopharmacology	1	7.05
Neuroreport	1	1.52
Neurosci & Biobehav Rev	1	8.80
Neuroscientist	1	6.84

Journal	Frequency	Impact factor
Oxford Uni Press	1	-
Percept & Psychophys	1	2.22
Perception	1	0.91
Phil Trans R Soc B	5	7.06
Phonetica	1	0.52
PLoS One	3	3.23
Proc Natl Acad Sci	1	9.67
Psychol Bull	2	14.76
Psychol Rev	1	7.97
Psychol Sci	2	4.43
Psychon Bull & Rev	1	3.37
Psychopharm	1	3.88
Psychophysiol	2	2.99
Q J Exp Psychol	3	4.67
Science	3	33.61
Time & Mind II	1	-
Trends Cogn Sci	5	21.97
Trends Neurosci	1	13.56
